# Hypermanganesemia with Dystonia Type 2: A Potentially Treatable Neurodegenerative Disorder: A Case Series in a Tertiary University Hospital

**DOI:** 10.3390/children9091335

**Published:** 2022-09-01

**Authors:** Khalid A. Alhasan, Walaa Alshuaibi, Muddathir H. Hamad, Suha Salim, Dima Z. Jamjoom, Aqeela H. Alhashim, Malak Ali AlGhamdi, Amal Y. Kentab, Fahad A. Bashiri

**Affiliations:** 1 Department of Pediatrics, College of Medicine, King Saud University, Riyadh 11461, Saudi Arabia; 2 Division of Pediatric Nephrology, Department of Pediatrics, King Saud University Medical City, Riyadh 11461, Saudi Arabia; 3 Division of Medical Genetics, Department of Pediatrics, King Saud University Medical City, Riyadh 11461, Saudi Arabia; 4 Division of Pediatric Neurology, Department of Pediatrics, King Saud University Medical City, Riyadh 11461, Saudi Arabia; 5 Department of Radiology and Medical Imaging, College of Medicine, King Saud University, Riyadh 11461, Saudi Arabia; 6 Pediatric Neurology Department, King Fahad Medical City, Riyadh 11525, Saudi Arabia

**Keywords:** hypermanganesemia, dystonia, chelation therapy, *SLC39A14* gene, movement disorders, neurodegenerative disorders

## Abstract

Importance: Hypermanganesemia with dystonia type 2 is a rare autosomal recessive neurodegenerative disorder characterized by the loss of previously acquired milestones, dystonia, parkinsonian features, a high serum manganese level, and characteristic neuroimaging findings such as bilateral and symmetrically increased T1 and decreased T2/fluid-attenuated inversion recovery signal intensity in the basal ganglia. This condition is secondary to a mutation in the *SLC39A14* gene. Objective: To present a series of three cases of hypermanganesemia with dystonia type 2, which was genetically confirmed secondary to a mutation in the *SLC39A14* gene, and to describe the treatment and clinical course in these cases. Design: A retrospective case series. Setting: University, Tertiary hospital. Participants: Three unrelated pediatric patients with hypermanganesemia with dystonia type 2, genetically confirmed to be secondary to a mutation in the *SLC39A14* gene. Exposures: Chelation therapy using calcium disodium edetate. Main outcome(s) and measure(s): The response to chelation therapy based on clinical improvements in motor and cognition developments. Results: All three patients were started on chelation therapy using calcium disodium edetate, and two of them showed an improvement in their clinical course. The chelation therapy could alter the course of the disease and prevent deterioration in the clinical setting. Conclusions and Relevance: Early diagnosis and intervention with chelating agents, such as calcium disodium edetate, will help change the outcome in patients with hypermanganesemia with dystonia type 2. This finding highlights the importance of early diagnosis and treatment in improving the outcomes of patients with treatable neurodegenerative disorders.

## 1. Introduction

Manganese is an essential trace element involved in different metabolic pathways. It acts as a co-factor for many enzymes, helps synthesize neurotransmitters, and is involved in neuronal and glial cell function [1]. Manganese homeostasis is regulated by SLC39A14, a manganese uptake transporter, which enhances manganese uptake into the liver from systemic circulation [1]. Mutations in the *SLC39A14* gene lead to the accumulation of manganese in the blood and subsequently in the brain, causing neurotoxicity [2,3,4].

Hypermanganesemia with dystonia type 2 (OMIM #617013) is a rare neurodegenerative, autosomal recessive disorder. Individuals with this condition exhibit high blood levels of manganese. The diagnosis is confirmed by detecting a pathogenic biallelic mutation in the *SLC39A14* gene [1]. Symptoms usually start between the ages of 6 months and 3 years, with delayed motor development or loss of acquired milestones, progressive dystonia, rigidity, spasticity, and parkinsonian features. Cognitive dysfunction is not a prominent feature; however, some degree of learning disability is present [1,5].

Patients with hypermanganesemia also have characteristic brain imaging findings, including high T1 signal intensity in the basal ganglia, specifically the globus pallidus, and less commonly in the cerebellum and brainstem [6,7,8]. Although these signal changes can improve with chelation therapy, they have been reported to persist despite treatment [4,9]. The pallidal index, which is defined as the ratio between the signal intensity in the globus pallidus and the subcortical frontal white matter on axial T1-weighted magnetic resonance imaging (MRI), is significantly higher in patients with hypermanganesemia and can be used as an indirect and noninvasive tool to assess disease progression [4].

Chelation therapy with calcium disodium edetate increases urinary manganese excretion and lowers its blood level. It also slows disease progression [1]. Here, we report three new cases of patients from three unrelated families who presented with motor regression and a progressive dystonia phenotype and were diagnosed with hypermanganesemia with dystonia type 2. We discuss the clinical presentation and effect of chelation therapy in these three cases. 

## 2. Methods

In this retrospective case series, we described the cases of three patients diagnosed with hypermanganesemia with dystonia type 2. The required ethical approval was obtained from the ethical review committee board at King Saud University, College of Medicine, Riyadh, Saudi Arabia (IRB number: E-22-0605). The parents gave their consent before starting the therapy. 

Their clinical data were obtained via a chart review of the patient database at King Saud University Medical City, Riyadh, Saudi Arabia. The patients were diagnosed using molecular genetic testing via whole-exome sequencing (WES). Once the diagnosis was established, the baseline serum manganese level was determined. Thereafter, the patients were administered regular chelation therapy at intervals of 4–6 weeks. Each therapy cycle consisted of 5 daily doses of calcium disodium edetate, administered at 1500 mg/m^2^ of body surface area (BSA) per day. Before and after each cycle, the patients underwent tests to determine the levels of manganese, copper, lead, iron, and zinc. 

### 2.1. Patient 1

A 5-year-old Egyptian girl presented with regression of milestones. She was born at term and developed normally until the age of 9 months. Thereafter, her motor skills started to decline and she started showing spasticity with abnormal dystonic movements. An analysis of her family history revealed that she had an older sister who passed away at the age of 14 months with a similar presentation, but her diagnosis had not been confirmed. 

At the time of presentation, the patient had severe hypertonia, spasticity with dystonic movements, and brisk deep tendon reflexes in all the limbs. She had no apparent dysmorphic features and used a wheelchair but had little social interaction. Her laboratory test results showed normal blood cell counts, normal electrolyte levels, and normal renal and liver functions. An MRI of the brain exhibited high T1 and low T2 signal intensity in both the basal ganglia, specifically the globi pallidi, associated with central blooming on susceptibility-weighted and gradient-echo images. She also had mild global brain atrophic changes. On the basis of these findings, she was suspected to have hypermanganesemia. The diagnosis was confirmed by genetic testing, which showed a homozygous nonsense variant in the *SLC39A14* gene, chr8-22408352-G-T (*SLC39A14*:p.E105*), previously briefly reported [10] is classified as pathogenic according to Clinvar and likely pathogenic according to the American College of Medical Genetics (ACMG) guidelines.

She was started on chelation therapy with calcium disodium edetate. The levels of manganese, zinc, copper, iron, and lead were measured before and after each session of chelation therapy. These measurements revealed that her manganese levels were remarkably lower after chelation therapy than before. Her initial plasma manganese level was 780 (ug/L), and after chelation therapy, it dropped to 55 ug/L. Unfortunately, the patient’s family returned to their home country, and she was lost to follow-up. 

**Figure 1:**
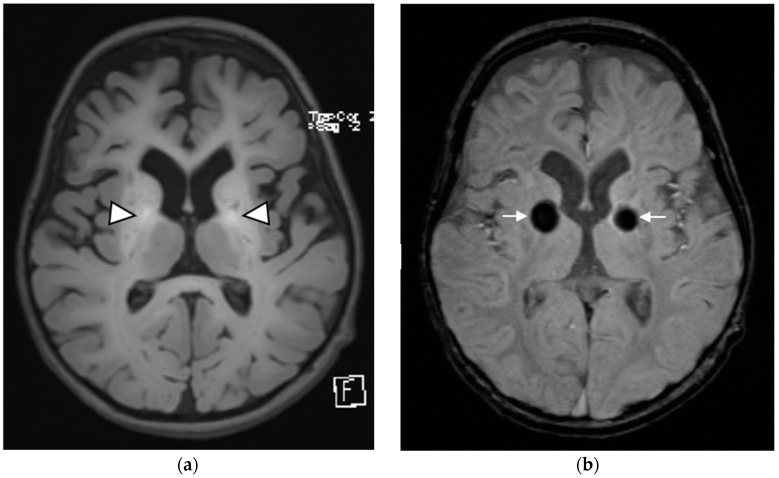
*Patient 1*.

Figure 1a Axial T1WI MRI of the brain at the level of the basal ganglia demonstrating bilateral symmetric T1 hyperintensity in the globi pallidi (white arrowheads). Figure 1b Axial susceptibility-weighted images show corresponding blooming artifact (White arrows). There is a mild degree of cerebral volume loss evidenced by enlargement of the ventricles and widening of the extraaxial CSF spaces.

### 2.2. Patient 2

A 13-month-old Yemeni boy presented to the pediatric neurology clinic with a history of developmental regression, worsening spasticity, and abnormal movements. He was born at term via uncomplicated spontaneous vertex delivery. He had acquired all the appropriate milestones, was sitting independently and babbling, and had appropriate social interaction. At 8 months of age, he began to show a progressive regression of milestones, developed worsening spasticity, and started to have abnormal dystonic posturing of the upper limbs. His parents were not consanguineous, and he had three healthy siblings. When he presented to the neurology clinic, he was unable to roll over and had difficulty feeding. Nevertheless, he demonstrated stranger anxiety and was able to recognize his parents and siblings.

**Figure 2:**
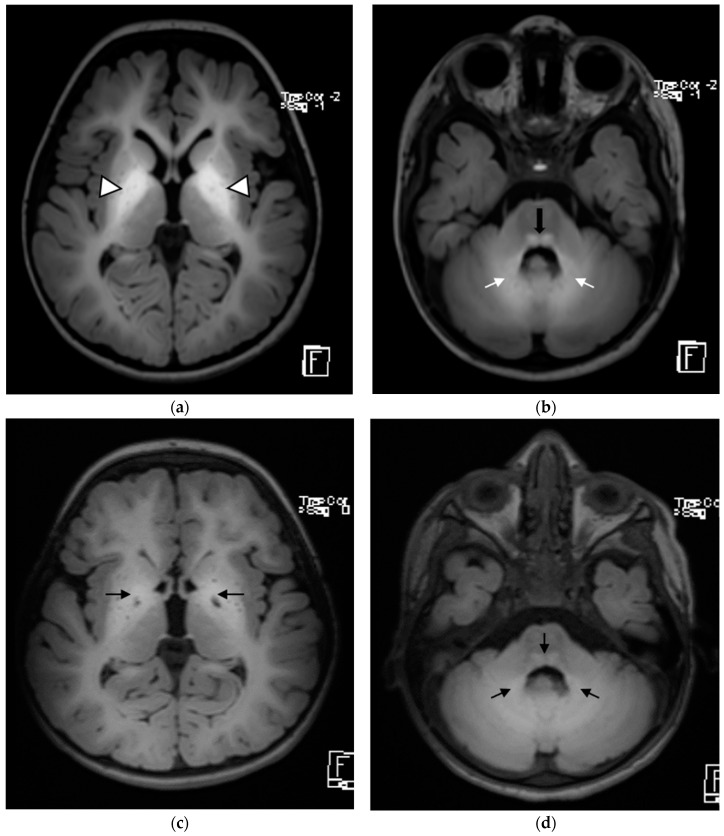
*Patient 2*.

A clinical examination revealed axial hypotonia with dystonia in the right upper limb. His deep tendon reflexes were brisk, and he had a positive Babinski’s sign and clonus in the lower limbs. His laboratory test results showed normal blood cell counts, liver and renal functions, and urine organic acid analysis values. An MRI of the brain revealed symmetrically increased T1 and decreased T2/fluid-attenuated inversion recovery signal intensity in both the basal ganglia, predominantly in the globi pallidi. Similar but less pronounced changes were noted in the cerebellar white matter and the posterior brainstem (Figure 2). Abdominal ultrasonography showed no abnormal findings. WES revealed a novel homozygous frameshift variant, chr8-22273308--AT (SLC39A14:p.E261Lfs*14), which is classified as likely pathogenic (class 2) according to the American College of Medical Genetics (ACMG) guidelines, which confirmed the diagnosis of hypermanganesemia with dystonia type 2.

The patient was started on chelation therapy at the age of 16 months, with 4–6 weekly cycles comprising 5 doses of calcium disodium edetate. His starting dose was 1500 mg/m^2^ BSA per day. Initially, each dose was administered over 16 h; however, in the second cycle, the patient developed a mild allergic reaction in the form of skin rashes. Hence, we prolonged the duration of infusion to 24 h, which was well tolerated. The levels of manganese, copper, lead, zinc, and iron were measured before and after each chelation cycle. We also checked liver and renal functions and blood cell counts at each cycle. He had completed 18 cycles of chelation therapy till the time of writing this paper.

Since starting chelation therapy, he has shown some improvement in his dystonia and spasticity. He has also been undergoing regular physiotherapy sessions, which have contributed to his recovery. He exhibited slow but steady progress in his development and could roll over and hold objects with his hands. His serum manganese levels also improved (Table 1). Brain imaging was repeated, and it revealed the same changes as seen on initial MRI at the time of diagnosis. No new lesions or extensions of the previous lesions were observed.

Figure 2 Axial T1WI MRI of the brain showing Figure 2a bilateral symmetric T1 hyperintensity in the basal ganglia, specifically involving the globi pallidi (white arrowheads). Figure 2b T1 hyperintensity is also seen in the dorsal pons (black arrow) and in the medial cerebellar white matter (white arrows). Figure 2c,d These signal changes have improved after initiation of chelation therapy (black arrows).

### 2.3. Patient 3

A 3-year-old Saudi girl was referred to the pediatric neurology clinic with developmental regression. She was born at term via spontaneous vertex delivery. She was healthy and achieved appropriate developmental milestones; she started walking by the age of 1 year, started running after the age of 2 years, and had appropriate fine motor and normal cognitive functions. At the age of 30 months, she started tiring out easily with activity and falling while walking. Eventually, she lost the ability to walk and subsequently lost the ability to sit unsupported. As the disease progressed, she began to experience dystonia, with prominent hand involvement associated with a flexed dystonic posture. She had been able to speak in a few sentences, but she started to stutter occasionally with disease progression. The parents were consanguineous but had no family history of similar presentations. She had one younger sister who was healthy.

An MRI of the brain revealed symmetrically high T1 signal intensity involving boththe globi pallidi and the dorsal midbrain and pons; both the superior cerebellar peduncles; and cerebellar white matter. However, no restricted diffusion or abnormal enhancement and no spinal cord abnormalities were observed. Her serum manganese level was 4900 ug/L (normal range, < 2.5 ug/L). The diagnosis was based on MRI findings and molecular genetic testing via whole-exome sequencing (WES), which revealed a homozygous missense variant, chr8-22273712-G-A (*SLC39A14*:p.G366S). This variant has been classified as a variant of uncertain significance (VUS), suggestive of the diagnosis hypermanganesemia with dystonia type 2. Segregation analysis showed the heterozygous status of both parents; this variant has been re-evaluated by a molecular geneticist in the context of the segregation analysis and high manganese level but still deemed to be VUS until more published or in-house patients are reported and well-established in vivo or in vitro functional data is available.

She was started chelation therapy using calcium disodium edetate at 1500 mg/m^2^ BSA per day, with 5 daily doses. At therapy initiation, she was found to have hypertonia, with brisk deep tendon reflexes in all the limbs. She also had mild intentional tremors. At the time of writing this paper, she had completed 9 cycles of chelation therapy. The treatment was well tolerated without any adverse events. On neurological reassessment during her last therapy cycle, she was found to have less hand dystonia and managed to open and close her fist. However, she had not made sufficient progress in gross motor development.

Table 1 summarizes the clinical, radiological, and laboratory findings of all three patients.

**Figure 3:**
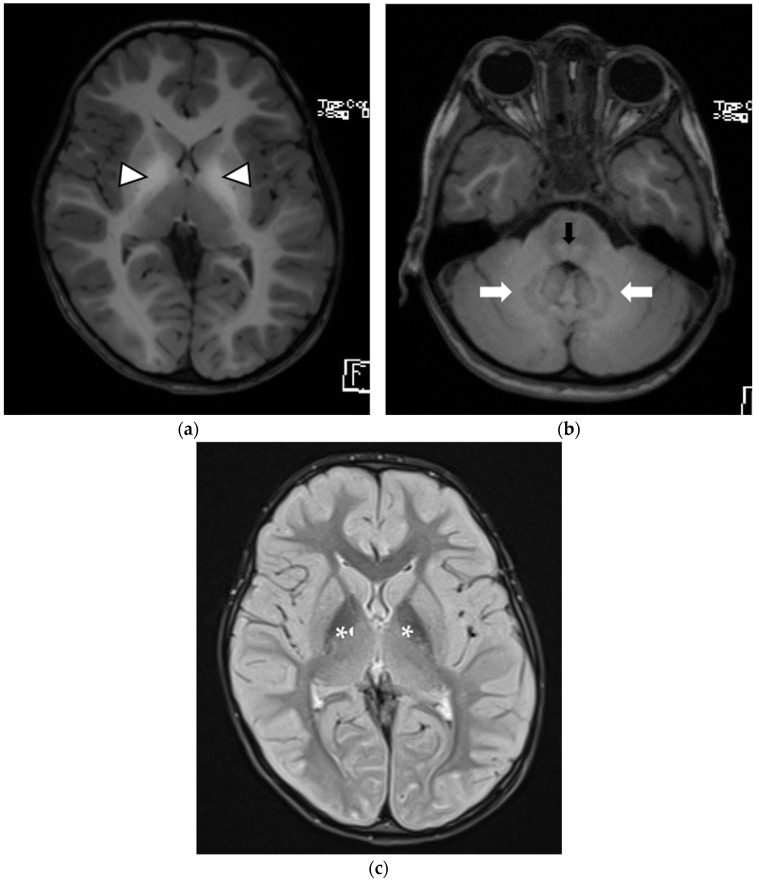
*Patient 3*.

Figure 3a,b An axial T1WI MRI of the brain showing bilateral symmetric T1 hyperintensity in the globi pallidi (white arrowheads), as well as the dorsal pons (black arrow) and cerebellar white matter (white arrows). Figure 3c An axial FLAIR image at the level of the basal ganglia shows corresponding hypointensity in the globi pallidi (white asterisks).

## 3. Discussion

We report the cases of three unrelated patients who presented with developmental regression and dystonia between 8 and 30 months of age and describe their clinical presentations and response to chelation therapy. All three patients were found to have a homozygous mutation in the *SLC39A14* gene, which led to the diagnosis of hypermanganesemia with dystonia type 2. 

Manganese is an essential element involved in numerous metabolic pathways, such as protein, carbohydrate, and lipid metabolism [11]. It is involved in providing stability to the immune system, ATP production and cellular energy, blood clotting, and bone and connective tissue growth [12]. It also acts as a co-factor for many enzymes, some of which are involved in neurotransmitter synthesis. The intracellular levels of manganese are stably maintained through several mechanisms [13]. However, this manganese homeostasis is disrupted in patients with inherited hypermanganesemia, which is caused by pathogenic variants of transporter-encoding genes such as *SLC39A8*, *SLC30A10*, and *SLC39A14* [14,15,16].

All of our patients developed normally until disease onset, i.e., when they developed dystonia, spasticity, and hypotonia. Such a disease presentation has been reported in all other cases described in the literature [1]. Moreover, the patients did not have significant behavioral changes, which is typically seen in acquired manganese toxicity in adults, as opposed to the inherited form [17].

Tuschl et al. explained that the main function of the SLC39A14 transporter in humans is hepatic manganese uptake for subsequent biliary excretion through the SLC30A10 transporter. They also reported that SLC30A10 mediates the efflux of manganese in the liver and brain [1]. Mutations in the *SLC39A14* gene cause a defect in the hepatic manganese uptake, leading to an increase in the manganese level in the body and subsequent deposition in the brain [1]. There are 74 classified variants found in the literature in *SLC39A14* gene; 19 are pathogenic and mostly missense (source: Varsome database). No genotype–phenotype correlation was made; this might be related to the small cohort of patients. All of our three patients had the same phenotype as the patients reported before, such as patient one variant, *SLC39A14*:p.E105*, which was described before by Tuschl et al. [1] and Anazi et al. [10] with a similar presentation. The Patient 2 variant is novel as it has not been reported in the literature nor found in any public databases. The phenotype is also similar to what has been reported before. 

Interestingly, Patient 3 variant was classified as a variant of uncertain significance by Clinvar. However, a recent article by Montaser Namnah et al. [18] suggests this to be a pathogenic variant as it is predicted to damage the translated protein, leading to an increased level of manganese as we have seen in our patient. It is not clear yet why this patient presented later in life in comparison to our patient, who presented early childhood with more prominent features. Further research needs to be done to clarify the natural history of the patient with variants in SLC39A14.

The MRI features of patients included symmetrical T1 and T2 hyperintensity in the basal ganglia, where manganese deposition typically occurs and leads to parkinsonian features, such as bradykinesia, rigidity, and dystonia [13,19,20]. This characteristic pattern with T1 hyperintensity in the basal ganglia indicates manganese deposition and can help distinguish between hypermanganesemia with dystonia type 2 and other neurological disorders due to metal accumulation, such as neurodegeneration caused by iron accumulation in the brain. 

We measured the serum manganese levels in all patients, and all had elevated levels before chelation therapy. However, none had polycythemia or liver disease, as is typically observed in patients with *SLC30A10* pathogenic variants [20]. Chelation therapy with calcium disodium edetate has been shown to produce a good response, especially if initiated at an early age. The younger the patient, the better is the response to chelation therapy [1]. The same agent has been used in patients with *SLC30A10* pathogenic variants and has produced a favorable response [20]. We treated all our patients with calcium disodium edetate at 1500 mg/m^2^ BSA per day with five daily doses every 4–6 weeks. All patients tolerated the therapy well, except for the second patient, who developed a treatable allergic reaction. All patients showed a decline in manganese levels and showed improvements in their clinical examinations, except for the first patient, who was lost to follow-up after receiving only two doses. Given the genetic origin of this disease, chelation must be continued as a lifelong therapy to prevent further damage to the brain. 

In conclusion, inherited hypermanganesemia with dystonia type 2 is a rare genetic condition with devastating outcomes if left untreated. It has unique characteristics in terms of brain MRI and results in elevated systemic manganese levels. Treatment is readily available and cheap, with minimal side effects and very good clinical outcomes if started early. Performing the necessary genetic testing in suspected cases of hypermanganesemia with dystonia type 2 to confirm the diagnosis is of paramount importance in such treatable disorders because early diagnosis and management can yield good clinical outcomes.

## Figures and Tables

**Table 1:** Summary of the clinical characteristics, radiological features, and laboratory findings of the three patients.

	Patient 1	Patient 2	Patient 3
Age at presentation/Sex	9 months/F	8 months/M	30 months/F
Clinical features at diagnosis	Regression of acquired milestones Dystonia Spasticity	Regression of acquired milestones Dystonia Spasticity	Regression of acquired milestones Dystonia Spasticity
Age at diagnosis	5 years	16 months	3 years
Gene Mutation	*SLC39A14* rs879253764;NM_001351660.2):c.313G > T (p.Glu105Ter) Homozygous nonsense	*SLC39A14* c.776_777insAT NM_015359.6):c.776_777insAT (p.Glu261LeufsTer14) Homozygous frameshift	*SLC39A14* rs774860376; NM_001351657.2):c.1096G > A (p.Gly366Ser) Homozygous missense
Age at the start of chelation therapy	5 years	16 months	3 years
Brain MRI findings at the time of diagnosis	Symmetrical involvement of the basal ganglia, with decreased T2 and increased T1 signal intensity Mild global atrophy Mildly enlarged eye globes	Symmetrical involvement of the basal ganglia, with decreased T2 and increased T1 signal intensity, and the involvement of cerebellar white matter and posterior brain stem to a lesser degree	Symmetrical involvement of the basal ganglia, tegmentum of the midbrain, posterior tegmentum of the pons, both superior cerebellar peduncles and dentate nuclei, with decreased T2 and increased T1 signal intensity
Neurological manifestation before chelation therapy	Regular wheelchair use No social interaction Inability to sit without support No words spoken Generalized spasticity Brisk deep tendon reflexes Dystonia	Feeding difficulties Unable to roll over Able to recognize parents and siblings, and demonstrated stranger anxiety No words spoken Central hypotonia Bilateral lower limb spasticity Brisk deep tendon reflexes and sustained ankle clonus Dystonia in the right upper limb	Stuttering Unable to sit without support Preserved social developmental milestones Spasticity in all the limbs Brisk deep tendon reflexes Mild intentional tremors
Neurological manifestation after chelation therapy	Only 2 doses received, did not show significant changes	Decreased dystonia and improved spasticity Able to roll over (after 8 cycles) Started speaking few words	Improvement in hand function Decreased spasticity
	Patient 1	Patient 2	Patient 3
Manganese level before the first session	780 (ug/L) N: <2.5	119.2 (ug/L) N: <2.5	4900 (ug/L) N: <2.5
Manganese level after the latest session	55.2 ug/L	19.1 ug/L	n/d
Follow-up brain MRI	Not performed	Similar changes to those observed at initial MRI, with no new lesions and no extension of previous lesions	Similar changes to those observed at initial MRI, with no new lesions and no extension of previous lesions
Hepatic impairment with chelation therapy	None	None	None
Renal impairment with chelation therapy	None	None	None
Changes in trace mineral levels in the serum (copper, zinc, iron, and lead)	None	None	None
Total sessions completed	2	18	9

MRI: magnetic resonance imaging.
